# Toward Language Justice: Exploring Multilingual Captioning for Accessibility

**DOI:** 10.1145/3706598.3713622

**Published:** 2025

**Authors:** Aashaka Desai, Rahaf Alharbi, Stacy Hsueh, Richard E. Ladner, Jennifer Mankoff

**Affiliations:** Paul G. Allen School of Computer, Science and Engineering, University of Washington, Seattle, Washington, USA; School of Information, University of Michigan, Ann Arbor, Michigan, USA; University of Washington, Seattle, Washington, USA; Paul G. Allen School of Computer, Science & Engineering, University of Washington, Seattle, Washington, USA; Paul G. Allen School of Computer, Science & Engineering, University of Washington, Seattle, Washington, USA

**Keywords:** Captioning, Multilingualism, Language Justice

## Abstract

A growing body of research investigates how to make captioning experiences more accessible and enjoyable to disabled people. However, prior work has focused largely on English captioning, neglecting the majority of people who are multilingual (i.e., understand or express themselves in more than one language). To address this gap, we conducted semi-structured interviews and diary logs with 13 participants who used multilingual captions for accessibility. Our findings highlight the linguistic and cultural dimensions of captioning, detailing how language features (scripts and orthography) and the inclusion/negation of cultural context shape the accessibility of captions. Despite lack of quality and availability, participants emphasized the importance of multilingual captioning to learn a new language, build community, and preserve cultural heritage. Moving toward a future where all ways of communicating are celebrated, we present ways to orient captioning research to a language justice agenda that decenters English and engages with varied levels of fluency.

## 1 Introduction

Captions are a synchronized text representation of audio in prerecorded media or real-time communication, often used to provide access to those with receptive communication access needs such as d/Deaf and hard-of-hearing (DHH) and neurodivergent individuals [63]. A growing body of research in HCI is dedicated to understanding and improving captioning experiences (e.g., [6, 7, 27, 45, 64]). However, much of this research explicitly, or implicitly, focuses only on English captioning, erasing the needs of disabled people who communicate and consume content in “othered” languages. Over 50% of the world’s population is multilingual i.e., understand or express themselves in more than one language [40]. Even in the United States, about 67.8 million people speak a language other than English at home [30]. These statistics suggest a need to explore individuals’ captioning and accessibility experiences across different languages. Captioning is not the only technology with language disparities. Only about 500 of the world’s 7000+ languages are represented online, with English (and more recently, Chinese) dominating infrastructure and resources [108]. Speakers of these digitally disadvantaged languages are then themselves disadvantaged, with impacts on their communication practices as well as their adoption of technologies [114]. In addition to English centrism, researchers have called out monolingual bias present in technologies (i.e., supporting only one language), which stifles multilingual practices, such as switching between languages at the word, sentence, or document level [99]. Monolingual design is typical in current captioning interfaces as well: e.g., Zoom only offers automated captions in 35 languages (many of them still in *beta*) and only one at a time [118].

While research in natural language processing and speech recognition has begun to include low resource languages [88] and multilingual contexts [79], this research has not yet influenced the user experience of captioning, perhaps because these technologies are mostly not deployed. We argue it is necessary to understand how caption users are navigating variability in accessibility support across languages today. While some scholarship has explored captioning experiences in different monolingual contexts and regions [10, 32, 84, 85, 104], not much is known about how captioning experiences differ across linguistic contexts and how multilingual practices are represented by captioning. Thus, in this work, we sought to understand the current practices and challenges experienced by multilingual and disabled users of caption technologies – how can we design captions to support multilingual futures and all ways of communicating?

Given the intersectional nature of this work, we sought out theoretical frameworks that could help guide our analysis of the results [44]. In particular, we draw from the concept of language justice. Often used by interpreting agencies and community activists, language justice advocates for the right for everyone to communicate in their own language(s) and strives to create a world where no language dominates over any other – where we ‘communicate across differences without erasing differences’ [1]. Our analysis questions whether the goals of language justice align with multilingual caption users’ needs, and explores how multilingual captions can be a site of language justice.

We conducted a two-part study consisting of semi-structured interviews and diary logging with 13 multilingual and disabled caption users. Our participants represent a range of disabilities, and many languages were a part of their lives. Through elicitation of multilingual experiences in interviews and logs, participants reflected on multilingual accessibility – how it has been shaped by (un)availability of captioning, their comfort with the language, and sociocultural perceptions of access – and how it might be improved.

In this work, we contribute:

1. Empirical accounts of disabled captions users’ experiences navigating accessibility in a variety of languages, unearthing how fluency, linguistic affordances, and cultural perceptions impact caption design and adoption,
2. Descriptions of how participants’ practices of multilingualism – such as translation, transliteration, and translanguaging – are supported or hindered by captions, thus articulating what it means for captions to *be multilingual*,
3. Suggestions of how we might address existing inequities–offering both immediate directions for development as well as guiding principles for future work informed by language justice.

Overall, we argue for more integrated perspectives on language and disability in communication accessibility i.e., rethinking captioning as a language technology (as well as an accessibility technology). We believe this reframing allows us to recognize the multitude of ways language needs and access needs of users interact and collide with each other – highlighting the need to engage with language lives of caption users and decenter spoken English in the design of captioning technology. It also illuminates exciting new avenues for research, and we invite researchers and technologists to take up this work.

## 2 Related Work

In this section, we begin by giving an overview of empirical research on captioning. Then we discuss multilingualism and related terminology that is foundational to our work. Lastly, we highlight how languages and technologies are intertwined, outlining ways technology research reinforces ideas of monolingualism and English supremacy, emphasizing the need for multilingual captioning research.

### 2.1 Captioning Research in HCI

Captioning is defined as “the process of converting the audio content of a television broadcast, webcast, film, video, CD-ROM, DVD, live event, or other productions into text and displaying the text on a screen, monitor, or other visual display system” [80]. Captions can be generated by humans or by using automated speech recognition software. In addition to captions, which transcribe text in the same language as audio, subtitles are also prevalent in prerecorded media. These are translations to another language, sometimes referred to as interlingual subtitles.

#### Deaf and Disability Perspectives.

There is an extensive literature on captioning as an accessibility technology for d/Deaf and hard-of-hearing (DHH) individuals, including work on accuracy (e.g., [45]), design and display (e.g., [13, 64]), encoding additional information (e.g., [6, 26, 81]), and redefining metrics (e.g., [7, 54]), amongst others. There are also works that examine the adoption of captions in the wild and social dynamics that impact its use (e.g., [77, 94, 109]). Captions can also offer access to neurodivergent individuals, those with auditory processing disabilities, etc. However, research on captions with these populations is fairly limited. While research has more broadly explored neurodivergent individuals’ communication needs (e.g., [25, 52, 116, 117]) and established captions as an access support for some neurodivergent individuals (e.g., [4, 25])–captioning is the main focus for only a few works that include neurodivergent perspectives [76, 96]. Both works examine captioning of TikTok videos, including content creators’ [96] and consumers’ [76] perspectives. There is still a need to explore how captions support access needs of this broader group, and how they can be better designed to do so.

#### Language Perspectives.

A closer look at captioning papers published through 1994–2019 at CHI or ASSETS [71] reveals that English dominates given the geographical context for much of this work. While a small body of work has begun to explore different languages captions (e.g., French in classroom contexts [32], Greek in theaters [10], Chinese in augmented reality [84], Japanese in museums [85]) and accessibility in different regions (e.g., India [101], China [20]), there is not much discussion of how language or the broader cultural context shapes the practices of captioning. Takagi *et al*. [104] note the over 4000 characters in Japanese add complexity to real-time human captioning– highlighting that language can significantly impact constraints and affordances of captioning. Notably, advances in speech recognition are slowly increasing the availability of automated captioning on platforms (e.g., Google Meet, Zoom, YouTube, TikTok) – but the users’ perceptions of quality and practices of use have not been explored. Additionally, captioning has mostly been studied in the context of fluent users. Proficiency in language also impacts caption use – some research has explored how literacy impacts DHH individuals’ experience of captioning, noting that individuals with lower literacy are are less critical of technology probes [14], and examining how we might support reading ease (e.g., [55, 65]). There is room to further explore the impact of fluency on caption adoption and desirability.

Captioning has also been examined from a media studies and audiovisual translation perspective, such as captioning practices in TV shows and films. These works shed light on how to tailor captions for an audience that might not have linguistic access (i.e., through translations) or auditory access (e.g., subtitles for the DHH [2, 113]). A notable example is Gonzales’ case study of multilingual content creation [35], which surfaces unique considerations in captioning for linguistic accessibility vs. sensory accessibility and how they might inform each other. Other works of interest include those that explore captioning media containing multiple languages and study how to represent multilingualism in captions [89, 103]. However, the intersection of audiences that know multiple languages (at varying proficiencies) and have access needs has not been explored in depth.

### 2.2 Multilingualism and Related Terminology

There are over 7000 languages in the world [31]. Boundaries between languages, and distinctions between languages and dialects, are not objective but rather socially and politically determined [19]. There is a wide variation in the sounds or phonemes belonging to each language as well as practices of transcribing these sounds. The set of symbols used to represent the language is known as the script. There is not a 1–1 relationship between languages and scripts (e.g., English and French both use the Latin script, Hindi and Marathi use Devanagari, Japanese uses both Hiragana and Katakana, some languages are not written at all).

Multilingualism has been the study of many fields and has a range of definitions [23]. Roughly, a multilingual person is “anyone who can communicate in more than one language” [68]. Multilingualism can also be characterized from a societal perspective, where it refers to the ability of institutions and individuals to engage in multiple languages in daily life [23]. Notably, multilingualism does not require equal fluency in all known languages, and more so refers to an individual’s ability to use these multiple languages in everyday life [39].

Practices of multilingualism refer to how the multiple languages in someone’s repertoire come together in daily life. They may use **translation**, which involves communicating content from one language in another language while preserving or retaining meaning. They may use **transliteration**, which is the process of writing content in one language using the script of another language (e.g., transliterating Hindi in the Latin alphabet gives us Romanized Hindi). Lastly, they may be **translanguaging**, which often refers to the practice of switching between languages as needed (e.g., during a conversation). Translanguaging allows multilinguals to use their full linguistic repertoire and communicate without “watchful adherence to boundaries between languages” [82]. These practices of multilingualism are important in an increasingly globalized world – they support cultural diversity and expression of identity. They also weaken forces of language dominance and assimilation by allowing individuals to retain and share different languages [69].

### 2.3 Technologies and Language (In)justice

The world loses approximately 9 languages a year [95]. These language deaths are often guided by colonial violence, racism and ableism [62]. Technologies have played a mixed role in this endangerment and minoritization of languages spoken/signed by 94% of the world’s population [74].

On one hand, technologies make it possible for dispersed communities to connect with each other (e.g., with communication technologies), as well as access information and cultural resources in their own languages (e.g., with broadcast media) [67]. There is also a rise in translation technologies that make it easier for users to communicate and engage with content across language differences such as Google Translate which supported 243 languages in 2024.

On the other hand, technologies have also reified disparities. Resources aren’t equitably available or maintained across languages (e.g., Wikipedia [91]) and platforms only support some languages – thus digitally disadvantaging users of these languages [115]. Even with English, there are disparities in performance for different kinds of English, (e.g., biases against African American Vernacular English [61] or ‘accented’ speech [86]). Some technologies actively erase non-normative language practices: e.g., Bender *et al’s* work highlights how language models flatten variation and contribute to reifying linguistic hierarchies [12].

In addition to lack of support for certain languages, digital environments introduce unique considerations and constraints for practices of multilingualism. For example, researchers have unearthed biases in machine translation and language modeling (e.g., [46, 70, 92, 106]) and highlighted lack of support for certain scripts [8]. While some researchers have begun to explore multilingual users’ practices navigating these constraints (e.g., [78, 83, 100]), most attempts to make technologies ‘multilingual’ are disconnected from actual user practices [93, 99].

Within HCI, more and more researchers are exploring multilingualism and arguing for more multilingual research [48, 60]. In their work on non-native English speakers experiences with mobile phones, Karusala *et al*. propose an intersectional approach for language in HCI, that asks researchers to question “how users put diverse facets of identity through languages and the forces supporting or hindering their uses of language” [58]. Similarly, Cardinal *et al*. propose multilingual UX as generative cocreation [21].

However, not many of these works explicitly engage with language justice, besides a few exceptions [42, 43, 112]. Notable is Cardinal *et al’s* work, which focuses on how to move from language access to language justice in our community work [22]. Given that there are many ways language justice can be enacted through existing technologies, it would be promising to have it as an explicit goal guiding research and development.

### 2.4 Summary

For captioning technologies, current disparities in availability across different languages raises questions about how multilingual users navigate variability in support and what their access practices are in multilingual contexts. These multilingual contexts additionally bring variations in fluency and literacy, and thus also raise questions of how individuals’ language needs and access needs come together. There is an opportunity to understand how captioning design and use can be informed by language and culture, and how captioning might enact a language justice agenda.

## 3 Positionality

All members of the research team are multilingual, spanning languages such as Gujarati, Hindi, Arabic, German, Swiss German, Mandarin, French, American Sign Language, and English with varying fluencies from native speakers to learners. Some authors use captioning for communication access needs in different languages e.g., when talking to family and friends, watching films, scrolling social media videos, and meeting on remote video software for work. The authors experience in a multilingual world, and their encounters with non-English captioning for accessibility prompted their interest in pursuing this study. Prior to designing the methodological procedure, two authors spent about two weeks documenting the captions they consumed in different languages and how captions’ norms, format and (lack of) availability shaped their access needs. This reflexive exercise helped the research team develop informed questions while also keeping in mind how participants’ experiences may be different to that of the authors.

## 4 Methods

Given the dearth of research on multilingual caption users regarding their experiences in different languages, we aimed to understand the accessibility of multilingual scenarios, the availability of captioning, and how it might be improved. To do this, we conducted a two-part study consisting of semi-structured interviews, diary logs, and follow-up interviews.

### 4.1 Procedure

In the initial interview, we gathered the language background of participants to understand where and how often participants encountered different languages. Similarly, we asked participants about their disability identity, their access needs, and how captions supported (or sometimes hindered) these needs. We asked them to share and contrast accessibility of their experiences across different languages and in multilingual contexts. Particularly, we were interested in the role captioning played in these different scenarios, its availability, and quality. We also asked participants to comment on how culture shaped these access mediations in different languages with different people.

Following the initial interview, participants were invited to log their multilingual interactions over a course of a week. This diary study was designed to prompt deeper reflection on accessibility of multilingual interactions and elicit articulation of specific experiences from participants’ daily lives (rather than a broad overview). We sent participants specific instructions at the beginning of the logging period that outlined the different kinds of experiences that would count as “multilingual” (Table 1). These included personal conversations with friends or family, interactions with acquaintances online and in-person, social media consumption (vlogs, Instagram reels, TikToks) or entertainment (movies, TV shows) – any experiences that involve multiple languages or non-English contexts.

In these instructions, we also incorporated a personalized list of potential experiences for participants to try based on the initial interview. We also pointed to captioning technologies in the participant’s languages that they might be interested in experimenting with, e.g., video conferencing software that had introduced automated captioning in different languages [36, 37]. We sent participants a reminder every day to share logs over text or email. In each log, participants were asked to briefly describe the context, the accessibility of the interaction, and the availability of captioning. The logs were intended to facilitate recall in the final interview. Participants were asked to submit a minimum of 3 logs through the week. They could choose to condense their logs and send them at the end of the day, or send them as each experience occurred.

For the final interview, we created a customized set of questions for each participant based on their diary logs. For each logged experience, we probed them further to understand the degree of technological and sociocultural support present. We aimed to understand the unique features of available captioning, its quality, and the accessibility of the overall experience in multilingual/non-English contexts (e.g., how did you feel about two sets of captions being displayed in that video? or how easy was it to follow the language switches in that in-person conversation?). In contexts without captioning, we asked participants about other access practices they used. Participants were then called to reflect on accessible multilingual futures.

All study activities were approved by the institutional review board (IRB) at the authors’ institutions. All participants were compensated for their time and expertise through a gift card. The first author led and conducted all the interviews, sometimes while other members of the research team observed. All interviews were scheduled for 60 minutes.

### 4.2 Recruitment and Participants

Our work is motivated by discrepancies in the availability and accuracy of captioning across different languages. Since this discrepancy likely impacts all multilingual caption users regardless of their specific disability, we sought to gather a diverse set of experiences by intentionally expanding our recruitment from DHH individuals to also include neurodivergent and disabled individuals who used captions for accessibility. Our focus on captioning meant we sought individuals who had experiences with multiple spoken or written languages, rather than multiple sign languages.

We shared our recruitment messages across a variety of disability and multicultural mailing lists, Facebook groups, and community organizations. We also recruited through snowball sampling. This resulted in 13 participants who identified as caption users for accessibility (i.e., identified as DHH, neurodivergent, and/or disabled), and understand or express themselves in more than one (spoken or written) language. Of these, nine participated in the diary logging portion of the study and final interviews. On average, each participant submitted *μ* = 12.33 (*n* = 9) diary logs – and most participants logged more online experiences (i.e., TV, social media, phone and video calls)(*μ* = 8.375*, n* = 9) than in-person interactions (*μ* = 4.25*, n* = 9). To support anonymization, we describe the languages covered in aggregate in Table 2.

### 4.3 Accessibility Considerations

All study activities were conducted remotely. Interviews took place on Zoom, and diary logging occurred asynchronously. To support accessibility of the research process for both participants and researchers [72], we offered CART, ASL interpretation, translation, and a copy of questions ahead of time. Some participants preferred automated captions, in which case participants used Zoom captions and researchers used CART. Sometimes they used chat to type out specific examples in their languages (P2) or when they preferred to be voice-off for a portion of the interview (e.g., P1). Often participants and researchers both spelled out words that were unfamiliar to the captioner (e.g., ‘Peranakan’).

### 4.4 Analysis

Three of the authors met and analyzed the collected data over the course of several weeks, following a reflexive thematic analysis process [17, 18]. The first part involved reading different transcripts and coming together weekly to discuss points of interest and potential themes. Following this initial familiarization with the transcripts, we conducted an open coding pass. Finally, we clustered generated codes together to larger patterns (e.g., practices of translation, practices of language-switching, cultural dimensions of access) and one of the authors reapplied these higher level codes to all of the transcripts. We further refined themes through the writing process. Overall, each of the transcripts has been read by multiple team members and there have been several discussions throughout the process to iterate on the analysis.

## 5 Results

**Figure 1:**
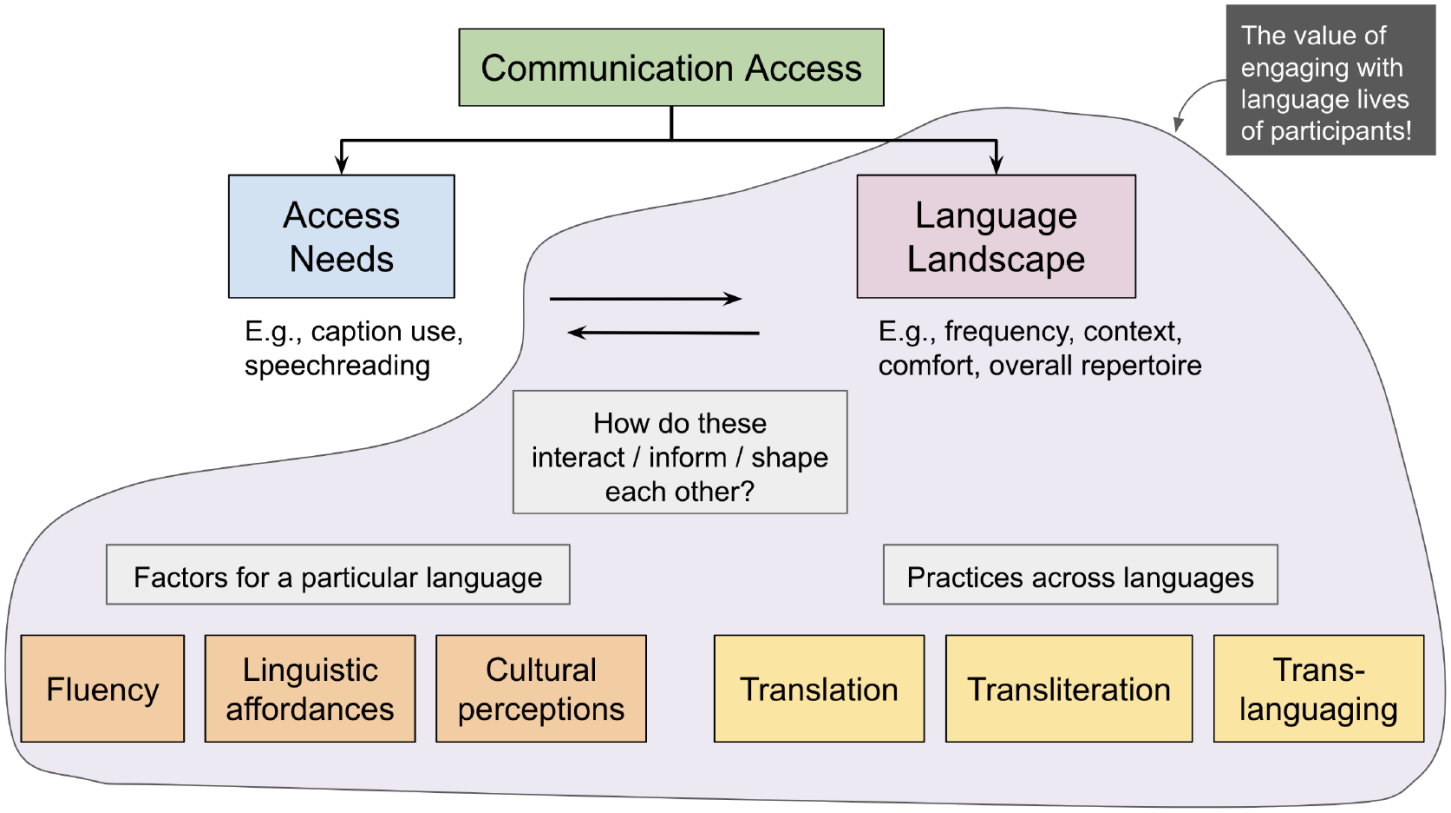
A visual map of the results section and our contributions. We illustrate the value of holistic approaches to communication accessibility by highlighting the the many ways disability needs and language needs interact and inform each other.

Figure 1 offers an overview of our results. The top half of the diagram corresponds to section Section 5.1, where we begin by articulating two different sides of communication access for our participants: their access needs and their language landscape. Through the rest of the results, we illustrate how these two sides of communication access (i.e., disability needs and language needs) interact and inform each other, as seen in the bottom half of the diagram. Section 5.2 discusses the factors that impact a participant’s experience in a specific language– namely, notions of fluency, language characteristics, and cultural perceptions. Section 5.3 builds on these to explore participants’ practices across languages, discussing practices of transliteration, translanguaging, and translation. Finally, Section 5.4 looks beyond a single experience to the systemic impact of (lack of) multilingual access on participant lives, thus emphasizing the need for holistic approaches to communication access that integrate language and disability perspectives.

### 5.1 Communication Access: Considering Both Disability and Language

In this section, we begin by describing the language journeys and accessibility needs of our participants, noting how their relationships with different languages and access often changed through time.

#### 5.1.1 Language Landscape.

The participants in our study discussed a variety of languages during the interviews. Since all were in the United States or Canada, they were comfortable with English (except for P3, who preferred ASL). In discussing the different languages they could understand or express themselves in, we found that participants rarely claimed to “know” a language. Instead, they often discussed when they learned the language, their comfort with different aspects (reading, writing, signing, speaking, understanding), how often they encountered it, and in which contexts.

Participants’ language repertoires were often broad and varied (e.g., P2: Korean, Spanish, ASL). The number of languages discussed also varied, with some, like P4, discussed two languages, whereas others, like P5, discussed seven languages. For each of these languages, degrees of comfort with receptive or expressive aspects differed, often shaped by how they learned the language and how they use it today. For example, participants who learned a language from their families only at a young age (e.g., P8: Mandarin, P10: Hindi) were sometimes less confident about reading and writing than those who learned it at home and school. Discussion of literacy was further complicated by numerous scripts available for a language (e.g., P9 could read Serbian^1^ in Roman alphabet but not Cyrillic). Additionally, sometimes shared histories between languages meant participants could understand a language they never learned (e.g., P4 understands both Brazilian Portuguese and European Portuguese).

Participants’ comfort with a language also changed over time and context, oftentimes with their migration patterns. For example, P2 grew up speaking Korean in South Korea, and only learned English after moving to the United States in middle school. Now, in their mid-twenties, they easily navigate the world in English, yet they feel like they are *“losing Korean”* (P2). Similarly, P12 told us, *“I guess [Hebrew] is my first language, technically, but since moving to [North America] with my family, we didn’t really speak it at home […] it’s definitely not at a very comfortable level anymore.”* (P12). One participant noted that an emerging fluency of a language is often neglected in Western spaces. P11 explained, *“ [Many people’s] understanding of how language works is so off-base, […] they say, okay, you speak French, you should be able to say every word in the entire dictionary, like, you know it, and it’s like – that’s not the way language works.”* (P11).

#### 5.1.2 Access Needs.

Our participants included d/Deaf and hard-of-hearing, neurodivergent, chronically ill, and disabled individuals. Some of our participants identified with more than one of these disabilities and some chose not to disclose specific diagnoses (as seen in Table 3). Therefore, in this section we discuss their communication access needs instead and *how* they use captions to meet these needs.

Many participants mentioned the need for visual components (such as seeing faces and bodies for lip movements and body language) to *speechread* [56]. Speechreading allowed participants to guess what was being said based on lip movements (bottom-up) and on context (top-down). Speechreading often coincided with caption use for our participants, as noted in prior literature [29]. Other access practices included limiting background noise and other sensory input (such as fans, bright lights, or heat). Overall, our participants shared a preference for written modes of communication.

Our participants used captions in a variety of contexts: in-person conversations, video calls, media, and at school or work. Table 3 lists some examples of scenarios where they used captioning from their diary logs and interviews. While some DHH participants could request CART as an accommodation at school or work, those with other disabilities were unable to since *“they want you to have a [specific] diagnosis”* (P11).

Captions played different accessibility roles: sometimes they were central to accessing incoming information, sometimes they played a tangential role. For example, for P2, captions were a backup: *“sometimes, I can’t really tell what the person was saying. I refer back to the captions”* (P2). For P5 and P6, captions additionally acted as a filter to *“focus on the sound that I’m actually trying to pay attention to”* (P6) and *“[provide] scaffolding for the auditory processing”* (P5). For P7, captions helped hold onto conversational threads and acted as a memory aid: *“retaining information or remembering prompts can be challenging, so I’m actually actively going back to the captions and looking at what you asked for.”* (P7).

Often captions played multiple roles for the same participant, depending on their needs in the moment and the communication context. In the absence of captions, participants would either disengage or *“struggle through”* (P5), depending on fatigue and importance. Table 3 lists some examples of multilingual scenarios where participants did not use captions, either due to personal choice or due to a lack of availability.

In the following sections, we discuss how different factors for a particular language and across languages impact these roles and shape how captions offer access.

### 5.2 Factors that Impact Communication Access

In this section, we discuss how participant experiences of access are shaped by their comfort with a language, its linguistic affordances, and cultural context. We highlight the impact of these factors on the design and use of captions, demonstrating that 1) we need to move beyond English captioning as the standard and 2) language and culture need to be foregrounded in captioning research.

#### 5.2.1 Communication Access Conditioned by Fluency.

As shown in Section 5.1, participants have varying levels of fluency across different languages and different types of proficiency within the same language. This provides a rich context within which participants have developed their communication access practices. For example, less familiar languages might demand a slower pace of communication, resulting in participants asking conversation partners to slow down or slowing videos while watching them. For P3, for whom English is their fourth language, *“if we are conversing in English, I need to be a lot slower and they need to be slower with me.”* (P3).

Language proficiency can additionally affect speechreading ease. Participants noted that strong knowledge of vocabulary (necessary for top-down speechreading) and the ability to recognize mouth patterns (necessary for bottom-up speechreading) are both crucial for effective speechreading. A lack of familiarity with key vocabulary relevant to the communication context can thus make speechreading difficult in certain languages. For example, P9 watches old Serbian movies with parents and finds *“archaic Serbian”* (P9) hard to follow. Similarly, P10 sometimes doesn’t understand what her grandmother said in Hindi, and attributes it to generational differences in phrases and vocabulary.

While captions can address some of the above concerns, they bring up their own considerations regarding fluency – notably, literacy. Being comfortable with speaking and listening to a language does not necessarily mean comfort with reading it. For languages with scripts novel to participants, reading proficiency had to be attained before captions could offer access: *“[My] confidence with reading [Arabic and Ukranian is] not as strong, so having captions in that language are less accessible or take more time or more energy to parse.”* (P5). Not only do captions require familiarity with the script or writing system at hand, they also require a reading pace that matches incoming speech. For example, even French, which has the same Roman alphabet as English, presents different access needs for P10: *“when I was in France and they wanted to watch more fast-paced stuff […] which I could read [the French subtitles, but] it was a bit tougher, so we would have to watch, like, comedies, or like just change the subtitles to English.”* (P10).

In the examples above, participants’ lack of fluency or proficiency in language directly affects how they request access or use captions – whether by slowing it down, relying on it more, or avoiding it until literacy is obtained. However, the need for access is not limited to those who lack language fluency or proficiency. In fact, P5 noted feeling like they had more access needs in their native and most familiar language (English) than the other languages, because they are not afforded the leniency often given to those learning a new language: *“Honestly, I think it’s … the overarching sort of background information of ‘you’re a language learner’ versus, like, ‘I expect you to be able to communicate fluently’, I think that is a very helpful – at least as far as getting access needs met”* (P5). Communication partners were more willing to alter conversation pace and fluency and be responsive to requests for rephrasing and other access needs when talking to a language learner, rather than someone assumed fluent. This sentiment was shared by P11, who felt there was less stigma for accessing support (such as captioning) in other languages compared to their native language (English), *“I guess there’s stigma, and it feels like – I’m obviously an English speaker, why can’t I just hear what you’re saying?”* (P11).

These stories show how normative assumptions about fluency impact use of captions *“if you want to be considered fluent in the language, you have to be able to hear it properly, but there are people who can’t hear languages properly”* (P13). This raises questions about who is included in the conception of a typical caption user and in what contexts.

#### 5.2.2 Communication Access Molded by Linguistic Affordances.

Our participants’ perceptions of communication accessibility in a language did not only depend on fluency, but also the *affordances*, or features, of the language itself. For example, while speechreading, P4 found Portuguese speakers moved their lips a lot more than English speakers, making it easier to speechread. Other participants discussed different features of a language that make it feel easier to process or more accessible to them. For example, P11 found the rules for spelling and pronunciation for German made it *“really easy to picture the words, as they’re being said”* (P11). Similarly, for P5, *“being able to visually process language”* (P5) and not having to physically speak made ASL feel cognitively accessible.

Interestingly, the match between speech and writing system varies significantly from language to language. Participants remarked on how Spanish is written as it is pronounced (*“really transparent to the phonemes”* (P6)) but other languages such as French and English have less phonetic spelling, which impacts caption readability. P13 discussed Cantonese at length, explaining how writing system impacts descriptiveness of captions: *“there is quite a big difference between what the [captions] say and what is actually being said because spoken and written Cantonese are very different. [… the captions] are usually the more formal version, as opposed to what they say on screen which is a lot more casual.”* (P13).

The disparity between speech and text in Cantonese is because of the existence of a standard writing system across all Chinese languages^2^ (Mandarin, Cantonese, Hokkien in our participant pool amongst others). This standardized writing system allows written Chinese materials to reach a wider audience. It also support disambiguation of spoken dialects in media with the addition of standard captions. However, this practice of written standardization removes aspects like slang and intonation that are unique to spoken dialects. P13 shared, *“Having subtitles that are a more standardized version of what they are saying is helpful, but at the same time you know it’s not exactly one-on-one with what they are saying on screen, so that kind of sucks in terms of accuracy.”* (P13).

The above stories highlight the degree and type of information captions encode varies language to language. These linguistic affordances then bring up questions about the inherent ambiguity in captions – if not speech, what do they offer access to? They also call us to reflect on caption metrics like accuracy that may be ill-defined.

#### 5.2.3 Communication Access Shaped by Cultural Perceptions.

Participants discussed how certain cultural norms shaped perceptions of disability and accessibility. For example, P7 found that the widespread availability of Chinese captioning on media allowed them to recognize captions *“as a norm that provides access to a lot of different users […] I knew it was a tool that I could use for myself, even if I wasn’t, you know, fully interested in it at the time.”* (P7). They found that this norm was lacking in English-produced content.

Other participants offered contrasting experiences: *“I think Western culture is a lot more understanding […] Indian culture that I’ve experienced is a little more, like, defensive […] like, you’re kind of treated a little bit like a nuisance if you ask for accessibility.”* (P10). These perceptions of accessibility impacted participants’ adoption of captions – such as whether they would feel comfortable asking someone to turn on captions while watching media together, if they would openly use a captioning technology in a conversation, or whether they would request captioning as an accommodation.

Cultural norms also influenced how participants thought their access needs would be received. For example, P2 discussed norms of fast communication in Korean: *“Koreans they’re just going, doing, and eating, and working just fast and quickly through everything without a break. So, whenever I do ask, you know, can you repeat that or […] ask people to slow down. I do feel a little more self-conscious.”* (P2). These norms impact whether participants disclose their access needs and thus the role communication partners play in facilitating access e.g., whether they would work collaboratively to make captions work better by slowing down and monitoring errors.

Some participants developed ways to address inaccessibility within specific cultural contexts. P12 was often more covert in expressing their need in Russian, *“I just make light of it, like you know, make a joke around it and it helps the person slow down if I need a repeat.”* (P12). This desire for covertness impacted whether they chose to use captions or speechread, since speechreading is less visible and reduces the risks of disclosure.

### 5.3 Communication Access Transformed by Practices of Multilingualism

**Figure 2:**
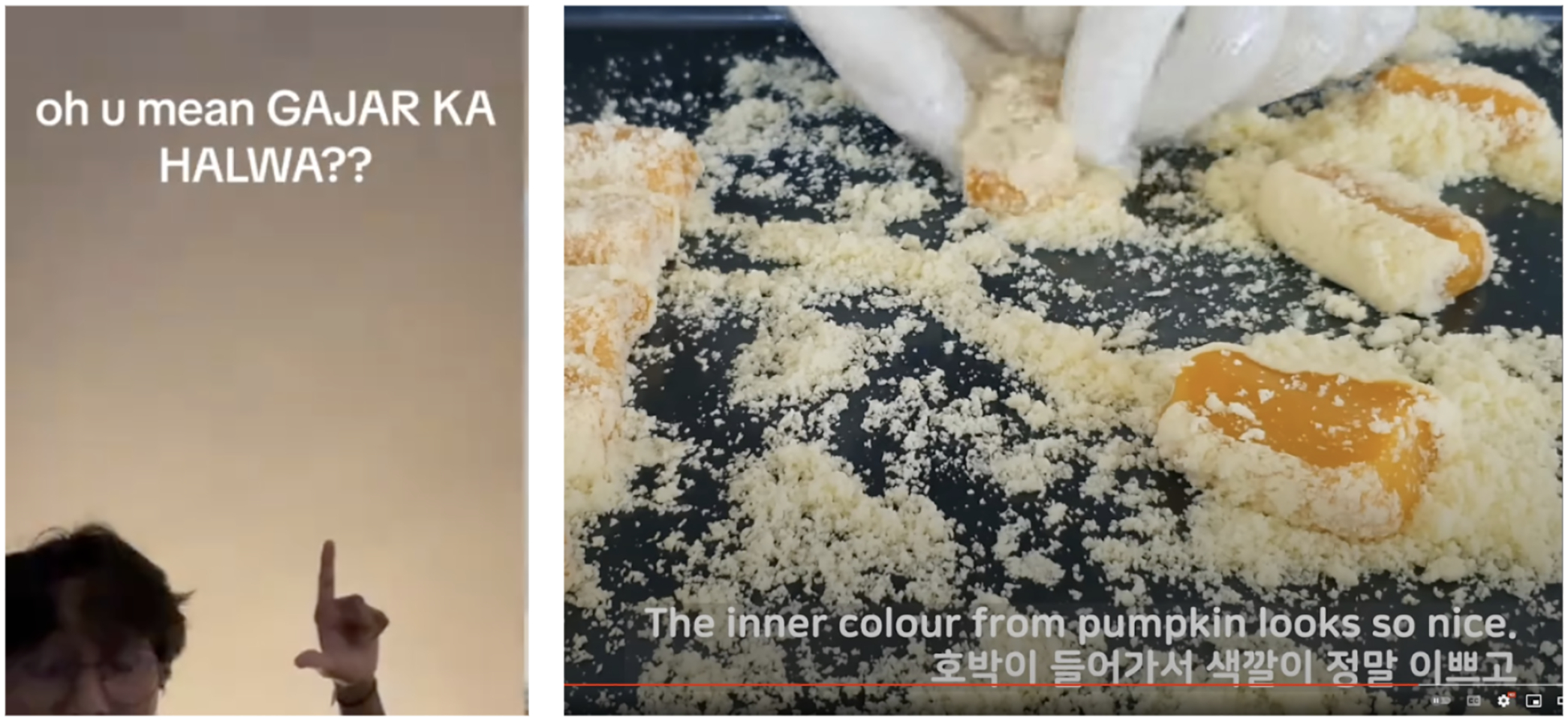
Practices of Multilingualism in Captions from Diary Logs. Left: Screenshot of captions from a video where both English and Hindi were spoken. Captions encapsulate both translanguaging (dynamically moving between languages) and transliteration (converting written script from one to another). The first half of the caption is in English, the second half is in Hindi, corresponding to the audio. The Hindi portion is transliterated to Latin script. Right: Screenshot of captions from a video where Korean was spoken. Two sets of captions are present: translated captions in English (top) and original language captions in Korean (bottom). While the video is monolingual (Korean), the presence of translated captions (English) introduce multilingualism.

Given our participants’ broad language landscape, the different languages they knew interacted in many ways. Here we discuss how three different practices of multilingualism—translation, transliteration, translanguaging—facilitated or hindered access provision for participants. We also trace how captions embody these practices of multilingualism (e.g., Figure 2) and how participants use captions to meet both language and access needs.

#### 5.3.1 Practices of Translation: Depicting Ideas Across Languages.

Translation played an important role in participants’ lives. While some participants often acted as translators for immigrant parents (P9), less literal translation was a key part of participants lives as well. When P10 and their partner watch Hindi movies together, P10 expands upon the English subtitles, using their knowledge of Hindi to evaluate quality and offer more nuance. In a conversation with Deaf friends signing ASL and a hard-of-hearing friend who was not comfortable with ASL, P3 *“interpreted into spoken Chinese from the ASL that the group was signing”* (P3) and vice versa. P3 was uniquely skilled to offer access in this situation – they know Chinese and ASL, but also needed their hard-of-hearing friend to repeat their spoken Chinese responses to meet their own access needs. These examples make clear the ways that language and disability collide – our participants often simultaneously navigated their access needs with regard to spoken language while using their multilingual skill to extend access to others.

Translated captions (or subtitles) were widely available, with media from different languages often captioned in English to increase reach and audience. For those familiar with the language at hand, accuracy of translated captions was sometimes questionable, making them prefer original language captions instead. Along with disagreements about intended meaning, participants found that translations did not conserve aspects that are key to the viewing experience: *“It’s either too literal of a translation or it’s just the feelings of the translation isn’t right. […] they use a lot of poetic phrases in Chinese, but when they translated it over it felt really flat and boring”* (P8). However, many participants were driven to use these translated captions as they were the only option.

When participants were not comfortable reading a language but were familiar with the language, they used translated captions in interesting ways. For example, when watching Hindi media with English captions, P10 found themselves reading the English translated captions and mentally translating it back to Hindi to match the incoming audio. Similarly, P7 could read simplified Chinese and not traditional Chinese. When they encountered a Mandarin video with traditional Chinese captions, they opted for the English captions and made *“a few mental jumps […] translating from the speakers [voice] to English [text] and then from English back to Chinese”* (P7). This process allowed them to leverage their full linguistic repertoire and experience all aspects of the media that may have been lost in translation. In choosing type of captions, participants made a calculated decision between the cognitive demands of reading a less familiar language and cognitive demands of this internal translation.

#### 5.3.2 Practices of Transliteration: Adopting Different Scripts.

Transliteration is the process of writing words of a language in the script of a different language. For example, Figure 1 (left) includes captions with the words ‘Gajar ka Halwa’ which is a Hindi phrase^3^ transliterated to the Latin script. Participants encountered transliteration of this kind often in user-generated digital content including textposts, music videos, and open captions on TikToks or reels.

Some participants, like P10, appreciated transliterated captions as they offered access without requiring familiarity with a certain script or literacy in the spoken language. While transliterations removed the need to learn a specific script, they introduce new challenge – sometimes they had to sound out the transliteration to recognize what it meant. Transliteration also introduced a lack of spelling conventions. This hampered reading speed and sometimes comprehension, requiring participants to slow down a video or *“look in the comments to figure out what that means.”* (P12). As captions are ephemeral, this impacted the desirability of transliterated captions.

In addition to supporting users with low reading literacy, transliterations aided the process of language learning – such as making pronunciations of unfamiliar words clear, and helping learners familiarize themselves with a new script. However, some participants could not imagine using it outside of a language learning context, or preferred to master the original language script instead: *“Studying so many different languages I can get quite turned around on what sounds certain letters are supposed to make […] yeah, learning a new alphabet is hard, but after learning the alphabet that a language uses to transcribe itself, like, there’s a lot more confidence on my part of, like, what things are supposed to sound like or what things are supposed to be.”* (P5). Transliterations also present ambiguity – each language has its own phonemes, and a script different from the original may not be able to depict all the sounds in that language. Reading a transliteration may then be more confusing and require more processing power than reading text in its original script.

#### 5.3.3 Practices of Translanguaging: Moving Between Languages.

Translanguaging, the practice of fluidly moving between languages, allowed our participants to fully combine their linguistic resources. Language switches were sometimes motivated by the need to use specific vocabulary (e.g., P4 switches to English when having technical discussions at work) and often allowed them to foster greater understanding. This practice was not just limited to spoken languages, – for example, when P3 chats with friends who also know ASL and Chinese, they often find themselves fingerspelling Chinese words phonetically in an ASL sentence to refer to a specific concept in Chinese. This practice of translanguaging also supports learning – for example, P2 discussed how he supported his partner who was learning Korean: *“There’s like similar pronunciations from Spanish to Korean. If phonetic English pronunciations are not correct for Korean, we’ll translate it into Spanish phonetic pronunciations and it will sound a lot better.”* (P2). This pooling of resources across languages shows the commitment to collaborative meaning making rather than prescriptive rules of language use.

However, translanguaging is not without downsides – some of our participants found it difficult to follow language switches and identify languages. To navigate this potential inaccessibility, P1 requested multilingual conversation partners to stick to one language during the course of conversation. When jumping into a conversation, P1 used his own responses to clarify the language being used, and sometimes started speaking first to set the language of the conversation. Another participant, P4, took a more direct approach, where he asked conversation partners to clarify whether they are speaking in Portuguese or English when he had a hard time following. This difficulty fluctuates given how expected the language switch is and given how the language and words have been merged together – borrowed words are common in many languages and these often take on new pronunciations in that language: *“[it is easy when] Portuguese pronounce it in a Portuguese way. When it is entirely new [word] […] I have trouble with that.”* (P4).

Participants found that captioning performed in disappointing ways when multiple languages were present in a piece of media or in a live conversation. For movies or TV shows, human generated captions often depicted the secondary language by name only e.g., [SPEAKS SPANISH]. This approach offered access to neither the phonetic nor the semantic content, making participants frustrated: *“I’m like, so what am I supposed to glean from this?”* (P9).

While some participants understood the choice to not translate might be from an artistic standpoint, e.g., the creator did not intend the audience to understand what was being said in that segment, the choice to exclude both transliterations and translations was deeply frustrating: *“I’m like, excuse me, I’d like to know what they said […] – yeah, it feels insulting [to the] people speaking and the work and the art itself.”* (P10).

While participants were able to toggle captioning language in some contexts (such as Netflix), caption options in all languages had drawbacks, given creators’ assumptions of language background of intended users: *“I’m watching this Portuguese show and I have my captioning set to English. And whenever they talk English, the captions disappear because they assume since I put English captions I know English”* (P4). Here, creators are also making assumptions about intended use of captions – to provide language access and not disability access. Some participants attributed this poor experience to not being able to choose multiple languages in captions. This is particularly relevant for automated captioning contexts where the inability to pick multiple languages for captioning caused captions to turn into *“completely gibberish”* (P13). However, this breakdown in captioning was helpful in indicating language switches in a video (e.g., P1 with YouTube). For those who used automated captions in in-person contexts, such as P4, they navigated this breakdown by manually switching the captioning language in their speech transcription app, a cumbersome process. They mentioned that the captioning software *was* capable of detecting language switches automatically – just not for all languages, highlighting inequitable development.

#### 5.3.4 Reimagining Multilingual Captions.

We asked participants to imagine accessible multilingual futures and reflect on what they would like to see changed. Many participants desired widespread availability of captions for all contexts, including media and real-time conversations. For automated captioning, they hoped for better transcription quality and increased accuracy in automatically detecting language switches. In absence of further technical development, they hoped content and media creators would invest in a *“quality assurance”* (P9) process.

Particularly important to participants was the addition of form factors that improved the multilingual accessibility of captions – *“an important metric is how well it handles multilingual settings.”* (P4). Some small scale changes with large potential for language switching contexts included indicating the language alongside the transcription *“in brackets above”* (P11) (e.g., [FRENCH] Mais oui…) and allowing users to choose multiple languages for captions on a platform. They also proposed having *“variety of scripts available”* (P7) to support a range of fluencies / literacies. They envisioned being able to use captions from different languages simultaneously – *“show both the translation and the original on the same caption bar.”* (P13). This would allow users to leverage information from multiple captions at the same time *“instead of having to open up a new tab, […] I could look at a translation right away.”* (P2). Users could choose which set of captions to focus on moment to moment, based on their comfort with the language, fatigue, and intention.

Participants also sought greater customizability for how language switches are handled and displayed – such agency to pick what would be transcribed vs. translated vs. transliterated based on their comfort in the moment. P13 pointed out, *“It would just be nice to have a lot more learning and accessibility options”* (P13). Customizability would reduce the assumption made about the language and disability background of the audience. Participants also brought up customizability of visual aspects like placement and font size. Even this desire was tied to multilingual fluencies: P10 for example, could catch background audio for Hindi media, *“wherever the captions are I’m fine, [… but] with Japanese I am a little bit more specific just because there’s more to [take in] for me. […] it would impact the size of the captions for me based on my familiarity with the language.”* (P10). Lastly, participants emphasized that these technological changes needed to occur alongside social changes. Some participants had experienced negative attitudes around caption use and emphasized the need to *“destigmatize captions”* (P8) as an accessibility technology. Education on *“how language works”* (P11) (e.g., fluency changing over time and context) would be crucial to address misconceptions and highlight the role captions play in language access as well.

### 5.4 Why Communication Access?

The previous sections have underscored the value of engaging with language in captioning by surfacing factors and practices. In this section, we present parts of our analysis that look beyond a single instance of (in)accessibility to the broader constellation of events, highlighting the systemic impact of (lack of) multilingual access on our participants’ lives. This allows us to bring to the forefront *why* communication access is important to participants, and *what* it affords access to. We unearth broader set of contexts and opportunities that participants desire access to – access to language, access to community and culture, and creating spaces where no language dominates or dies. We can see how their current use and vision of multilingual captions align with the goals of language justice.

#### 5.4.1 Multilingual Captioning as Access to Language and Language Learning.

Participants emphasized the importance of access to language. Languages allowed them to connect with their *“roots and heritage”* (P1), and learning languages was often necessary to navigate the public sphere (such as school or work or day-to-day errands). However, aspects of learning languages, like identifying new sounds and pronouncing new words were often inaccessible to our participants. Multilingual captioning played unique role in supporting language learning and thus affording them access to language.

For example, participants who were immigrants and learned English after moving to North America (P2, P9, P12) discussed watching TV shows for language immersion in early years: *“we ended up watching a lot of Star Trek of all things because it had English captioning.”* (P9). Similarly, P12 found themselves transitioning from using Russian subtitles (translated captions) to English captions (original language) as they became more familiar with English.

Interestingly, the role captions play shifts through their language journey. For example, P2 found that what captions offered access to changed over time – in earlier years they helped to distinguish the rapid stream of sounds from new language, and in later years they offered broader context, *“I think captions will always be helpful for me. […] I will say though, I think the more fluent you are with a language – least for me – I tend to use it less often.”* (P2). P7’s language journey with Hokkien was different – they were exposed to the language while growing up and lost touch in adulthood. They took a beginner’s class on Zoom to pick up the language again – unfortunately, there were no captions available. Remarking on the experience, they shared,*“I was a little bit ahead of the curve. So I did okay without the captioning but I imagine had I advanced to the third or fourth level, I probably would have needed access to captions.”* (P7). At an advanced level, small nuances in sound and speech would have been important to follow necessitating need for captions.

These examples highlight the dual role multilingual captions play in providing *both* language access and disability access against the backdrop of our participants’ language journeys.

#### 5.4.2 Multilingual Captioning as Access to Culture and Community.

As media offered participants an opportunity to immerse themselves in a language, it also offered a gateway to culture and community – only if the media was accessible to participants, *“language shapes your understanding of the world, right? And captioning [is] also a gateway to that.”* (P7).

Inequitable development of captioning across languages and poor quality of captions made participants reluctant to engage with non-English content. For example, for P13, *“a lack of captioning, or maybe a lack of me wanting to find the captions for French, has made me disconnected to a lot of the culture, a lot of the things that I learned.”* (P13). This exclusion from pop culture and digital media spaces had far-reaching consequences for community connection as well. P9 shared, *“Cause we don’t only watch media by ourselves. […] we might you know at home […] but it’s part of a conversation you’re gonna go ‘hey, have you seen this movie?’ and then if you can’t ever see anything like how will you feel like you’re close to those coworkers or family or friends?”* (P9).

Participants often learned languages to connect with their roots, or to foster deeper connection with friends, family, or partners. By offering/hindering access to language, captions could offer/hinder access to culture and community. For example, P7, who desired to connect better with their Peranakan Malay ancestry remarked on the lack of both original and translated captioning on shows uploaded to YouTube, *“while most people understand that we’ve experienced cultural loss and that younger generations do not have access to their Peranakan heritage and language, they [still] fail to provide translations. […] If I’m trying to follow along and I don’t understand what’s being spoken, it just feels difficult and makes it harder to access culture, especially when I’m already here in [North America] and not surrounded by community.”* (P7).

While the examples recounted above show how the absence of multilingual captions has negatively impacted participants’ access to community and culture, they also highlight the potential that thoughtfully designed captions have for fostering that access.

#### 5.4.3 Multilingual Captioning as a Site of Linguistic and Cultural Preservation.

Our participants were passionate about languages and actively sought out multilingual experiences. Their immense respect for how languages offered access to culture and community made them mindful of how language could be exploited for power. P5 shared, *“[Languages] are these really wonderful keys to, like, history and culture and identity. And as such have been politicized [… to] untether people from their, like, historical or ethnic or religious or cultural backgrounds and connections, like forced assimilation”* (P5). In light of this history, they cared deeply about ways to preserve language and culture, *“it’s important to the people and also it’s important to the language, to be able to maintain that presence and that sort of steadfastness in existence”* (P5).

Tracing these goals to captions, they discussed how captions often standardize variations in speech in the process of transcribing to written form (also seen in Section 5.2.2). P1 shared, *“Hindi is spoken so differently from different parts of the country and the world. […] the sound of the word, the way they say it, I think that is sometimes lost when it comes to transcribing”* (P1). They highlighted how standardization could have far reaching consequences by erasing minoritized ways of speaking. They mentioned this had already happened with the use of standard dialects on broadcast media, *“everyone starts to speak like on the TV and radio.”* (P11).

Nevertheless, participants highlighted ways multilingual captions could be a site of preservation. P11 discussed how more descriptive or phonetic captioning styles, that push the orthographic rules of the language to better match what is being said (or particularly, how it is pronounced), could preserve these linguistic and regional variations while facilitating access (e.g., with Quebec French transcribed in written Joual^4^) – *“Personally, being a descriptivist with language I think it is really important to keep the diversity.”* (P11). Other kinds of multilingual captions, like transliterations and translations both bring tensions and opportunities. As we established in Section 5.3, translations may miss cultural context, and phonetic captioning styles like transliterations might be hard to parse. But participants did like the potential for linguistic preservation, especially in contexts like music or comedy, which were central to culture.

#### 5.4.4 Besides Multilingual Captioning.

In highlighting participants’ current uses and visions of multilingual captions, we have demonstrated participants’ hopes for the *outcomes*^5^ of communication access: access to language learning, access to community, and preservation of cultural heritage. By bringing these hopes for outcomes of access to the forefront, we show that multilingual captions are only one of many tools for achieving these outcomes.

For example, some participants had doubts about whether captions could facilitate access to language. Limitations of captions as a medium, particularly for languages with mismatch between writing and speech – *“spoken Cantonese and written Cantonese are quite different”* (P13) – made participants hesitant about caption use. Similarly, P4 found that access to Portuguese was better facilitated through speechreading, *“[T]he thing is, my lipreading much better in Portuguese”* (P4) and thus did not use captions with family.

While multilingual captions *could* foster access to community, some participants did not think that the dearth of captioning had impeded their access to community. P3 had *“learned how to work in the world without a lot of accessibility. […] Captions really are recent and new thing for me.”* (P3). They were used to using other access practices like writing notes or asking for repeats. Other participants (P1, P2, P3, P5) sought community and cultural connections outside of the ones they were born into by learning sign languages like ASL. P2 remarked, *“I wish more hearing people would recognize the rich culture that there is within sign language and Deaf [communities]”* (P2). This adoption of sign languages indirectly facilitated preservation, given the history of oppression of sign languages amongst audist^6^ world.

Thus, when we look at captions as a *“gateway”* (P7) to different activities and contexts, we can recognize that the use of captions is intentional and goal-driven. These intentions may change over time and context, and sometimes other access technologies and practices are better suited for reaching these goals.

## 6 Discussion

Our results highlight the many ways our participants navigate access in a multilingual world. We articulate notions of multilingual accessibility grounded in our participants’ lived experiences – particularly, their language lives, which are often lost in prescriptive ideas of what communication should be.

Our analysis showcases the importance of integrating language perspectives into captioning research and examining the interplay between linguistic and sensorial access needs. We underscore the potential for language justice – a practice that examines how language can be a tool for oppression *and* equity – to inform our work in this space. Building from language justice, we call researchers and technologists (1) to attend to factors like fluency, linguistic affordances, and cultural perceptions unique to each language/experience; (2) to foster practices of multilingualism such as translation, transliteration, and translanguaging in captioning; and (3) to reflect on and address the systemic impact of (lack of) multilingual access on communities, culture, and languages.

Informed by these results, we begin by commenting on the state of speech recognition research and offer some immediate directions for development. Looking further down the horizon, we discuss how we might change our approach to captioning research, thus offering guiding principles and preliminary spaces for inquiry. We conclude by discussing how language justice could be valuable beyond captioning research.

### 6.1 Immediate Directions

Addressing the disparities in availability and quality of captions across languages requires significant advancements in speech recognition technologies. For English speech recognition, deployed technologies and state-of-the-art models achieve single digit WER (Word Error Rate [34], which roughly corresponds to the number of errors in transcription for every 100 words). In contrast, the performance of monolingual models of other languages varies widely (e.g., 13–25 for Hindi [15], 16–22 for Japanese [57], 13–30 for Arabic [5]). Efforts to train multilingual models also yield disparate performance – for example, Whisper AI ranges from 5 to 80 WER (e.g., 5 for English, German; 15 for Chinese, Korean; 30 Hebrew) [87]. While NLP researchers are working on innovating new techniques, collecting better multilingual datasets (e.g, [53]), and exploring new metrics (e.g., [41, 59]), it will be a while before these developments trickle down to users and technologies are readily available.

In the meantime, we believe improving caption form and display is a promising avenue of work. Many of the experiences discussed by our participants relate to professionally captioned media (e.g., TV shows, movies) and user-generated content (e.g., YouTube videos, TikToks, Instagram reels). Our results point to several actionable directions for improvement (Section 5.3.4). Some could be implemented by revising captioning practices and increasing awareness, such as ensuring that the language is identified within the transcription in square brackets. Others require small changes to platform functionalities and leverage existing captions, such as allowing display of more than one caption at the same time (e.g., Korean on top, English at the bottom) and supporting selection of multiple captioning languages for multilingual media (i.e., videos with language switches). For these dual-track captions on multilingual media, we additionally recommend offering users more granularity in customizing font, size, and placement for different languages. Applying some automated approaches to transliteration could further allow for increased support for captions in multiple scripts (e.g., Simple and Traditional Chinese and Pinyin) and thus support users with different literacies. While these are low technical complexity, they would improve multilingual accessibility considerably.

### 6.2 Guiding Principles

In addition to NLP advancements and captioning design, we believe that to truly redress the imbalances we see across languages today, we require a fundamental shift in how we approach captioning research. Below we offer three guiding principles to reshape our ethos: centering language, decentering fluency, and recognizing fluidity of use. For each of these, we highlight how they inform our spaces for inquiry.

#### 6.2.1 Centering Language.

We argue language should be a crucial axis by which to design captioning technology, rather than an afterthought. English captioning has become the standard in HCI research, and an implicit assumption of generalizability reinforces the idea of “voice from nowhere” [93] – that there exists a neutral language and that language is English. In contrast, our work shows that captioning norms and practices are shaped by the linguistic, historical, legal, and structural processes inherent to languages (Section 5.2.2), and that we need to bring these contexts to the forefront. Doing so not only recognizes the *situated* and *specific* nature of our knowledge, but also allows us to begin building tools that support typologically diverse languages and their users.

For example, our participants discussed linguistic affordances like spelling conventions for German or cadence for Portuguese that impacted accessibility of their experiences. P13 discussed how *“spoken and written Cantonese are very different”* (Section 5.2.2), impacting what information captions can encode and convey. Additionally, language and culture are closely entwined, and our study further shows how cultural norms influenced access practices and adoption of captioning – such as P12 who felt the need to be covert expressing their needs in Russian vs. P7 who felt captioning was a norm in China. These results point to the value of language-centric perspective.

*How might this inform our spaces for inquiry?* One direction for research is extending work on caption style, placement, error display to different languages and exploring how language characteristics impact the efficacy of current best practices. For example, how do unique visual affordances, like vertical reading order and logographic script impact the use of visual space while captioning? Another question rises for languages where writing is significantly different from speech (e.g., Cantonese) – what do accurate captions (and thus metrics) mean in this context? A systematic exploration of how listeners of these languages navigate discrepancies can help us design captions that better meet their access needs. Yet another direction could aim to improve usability of automated captioning in these languages through human-in-the-loop paradigms like crowd-sourcing captions and correcting errors. These approaches have often been studied for English in Western contexts – exploring how language characteristics shape efficacy and cultural norms impact generalizability are open questions.

#### 6.2.2 Decentering Fluency.

In this work, we engage with participants’ experiences in a range of languages – from those they grew up speaking to those they were just beginning to learn. As a result, we could recognize the role proficiency and notions of fluency played in participants’ experiences with multilingual accessibility. This is a contrast to most research in our field, which often only engages with fluent (English) speakers as potential users of technology. We argue this implicit prioritization of fluency not only excludes non-native speakers of languages, but also ignores how fluency fluctuates over time. Migration, relationships, and globalization called our participants to pick up (and sometimes drop) languages in different periods of their lives. We call researchers to engage with these complexities of language use by decentering fluency.

Our participants discussed many experiences that occurred in less-fluent languages but were no less important to them – such as learning languages with their partner, watching TV shows with friends, and connecting to their heritage and families. Decentering fluency allowed us to unearth our participants’ “subjective, emotional relationships with languages” [66]. They further highlighted how fluency conditioned their use of captions (e.g., familiarity with script and reading speed) thus impacting access. Importantly, our participants highlighted how fluency as an ideology encodes ableism – consider P13’s quote in Section 5.2.1, *“if you want to be considered fluent in the language, you have to be able to hear it properly, but there are people who can’t hear languages properly”* (P13). These assumptions discount any non-normative communication styles. This is echoed in P11’s experiences with caption use in English, where she felt stigmatized as a ‘native’ speaker. By reducing expectations of fluency, communication and access provision became easier for our participants (such as P5’s experience with access needs being lower in a language learning context).

*How might this inform our spaces for inquiry?* A promising direction for research is actively building technology that supports users with varying fluencies. For example, given the complex interplay between literacy and caption use, how might we redesign captions to support novice readers? Beyond readability, there is also a broader question of supporting semantic understanding. We could extend research from audiovisual translation literature which largely focuses on analyzing captioners’ choices in subtitling media (e.g., [2, 9, 24, 89, 103]) to actually engage with the experiences of audience members who use captions for accessibility and may be multilingual themselves. Similarly, the field of language acquisition could offer valuable perspectives in balancing the sometimes conflicting goals of language learning and access/understanding in the moment. We might also explore how practices of making language accessible in books and other text (e.g., including footnotes and rephrasing content) could apply to captions.

#### 6.2.3 Recognizing Fluidity of Use.

Much of this work aimed to highlight the complexities that rise from the multilingual fluidity of the real world. We find that this fluidity extends beyond language (i.e., switching between languages) to the use of access technologies and practices as well. Instead of constraining themselves to a particular access technology such as captioning (Section 5.4), participants leveraged different technologies and practices as needed. Their choices were guided not simply by the availability of captioning, but also by *how* it would support access and *what* it would facilitate access to: language, community, and cultural heritage (Section 5.4). We argue that recognizing this fluidity is necessary to designing technology that is useful in the real world.

Our participants perform complicated calculus while negotiating both language access and disability access in multilingual contexts since no one set of captions offers full linguistic and sensorial access. Consider a context with language switches – original language captions presume fluency, and translated captions presume the hearing ability and disappear with language changes (Section 5.3). Participants’ practices of multilingualism, fluidly combining their linguistic repertoires for translation, transliteration, and translanguaging, demonstrate the need to to think beyond static, monolingual contexts, and recognize how multilingual captions may adopt to changing needs.

Recognizing fluidity in use also calls us to be expansive in our conceptions of users. While prior work on captioning often focuses on the experiences of DHH people and evaluates the efficacy of transcribing audio to text, we opted to include perspectives of neurodivergent, chronically ill, and disabled individuals well. Doing so allowed us to surface unique ways captions supported access – by acting as a scaffolding for auditory processing, a focus tool, and a memory aid (Sectioin 5.1).

##### How might this inform our spaces for inquiry?

The different roles captions play in provisioning access for different users raises the opportunity to explore how these functions may inform design and evaluation (e.g., metrics) of captioning technologies. Systematically exploring what motivates use and non-use of captions amongst other access practices in a moment could deepen our understanding of communication accessibility for DHH, neurodivergent, and chronically ill individuals. Importantly, this perspective opens us to research that attends to the fluidity of access and explores what motivates an individual’s choice of technology, modality, and language in a given moment.

### 6.3 Beyond Captioning

Through a deep exploration of multilingual captions, we have shown how language and disability shape each other and shape what access means. We invite researchers to explore these ties in broader accessibility research, beyond captioning.

In the last few years, accessibility research has been engaging with disability justice, a developing framework and activist movement led by disabled queer people of color. Disability justice principles have been used as a guiding lens to redesign captioning technologies, such as orienting hearing and DHH people to *collectively* attend to captioning errors [75]. They have also been used to subvert ableist structures in broader accessibility research [49, 50, 102, 110, 111]. We call researchers to incorporate language justice agendas as well, given how closely it aligns with disability justice. In fact, Sins Invalid, a foundational disability justice organization, argues that language justice *is* disability justice – both movements demand we move in mutual respect for all peoples, regardless of whether or how they communicate [51].

For example, consider research on DHH communication more broadly. Language justice allows us to engage with Deaf individuals who identify as minoritized language users, it centers the linguistic richness and cultural importance of sign languages independent of the access they afford [28], and it encourages to respect the multitude of ways d/DHH individuals sign or speak or gesture – the ways they understand and make themselves understood [47].

We believe there is value in taking a “language-first” perspective beyond captioning too. English also dominates research on web navigation [90], audio description [73], technologies for visual access [3, 16, 107], and alternative-and-augmentative communication devices [105] and more. Since languages may encode and describe color, direction, sound, and space differently, diversifying languages explored can deepen our understanding of practices for sensorial accessibility. We believe these differences across languages fundamentally shape how we communicate information and mediate accessibility interpersonally or technologically. Decentering fluency and recognizing fluidity of use is equally important in these broader contexts as well.

More broadly, we encourage researchers to document and investigate accessibility practices across languages and explore how multilingualism is transforming the design and use of accessibility technology. Given that practices of transliteration, translanguaging, and translation can be powerful ways to preserve languages, we call technologists and researchers to reimagine all of our systems to foster these practices.

## 7 Limitations

Getting a representative sample of as a large and dispersed population as “multilingual caption users” is difficult. As such, through recruitment we tried to capture experiences from people with diverse language and cultural backgrounds, hoping to articulate the complexity and nuances and space for future research. However, despite offering language interpretation, our research was limited to multilingual caption users who felt comfortable communicating in English or ASL, which means we did not include perspectives of a large number of individuals with valuable and potentially broader cultural insights. All participants were located in the United States or Canada. Thus, our findings may not be applicable to other geographical contexts. Additionally, languages represented in our sample are mostly widely spoken. We invite future research to engage with languages at risk of disappearing due to colonialism and other oppressive structures (e.g., Hawaiian).

We conducted our study at an exciting time in the multilingual captioning world. Improvements in speech recognition technologies are resulting in deployment of automated captions in different languages to various platforms. For example, videoconferencing platforms such as Google Meet began adding non-English captioning in mid-2022. As of June 2024, Google Meet supports 87 languages [38], over half of which were added after we began data collection. Our results are situated at a point in time where for most users, lack of high-quality automated captioning support in their (non-English) languages is a reality. However, the shifting technological landscape may succeed in addressing this disparity in availability. This increasing availability of multilingual captioning presents an opportunity for future work to investigate individuals’ adoption and long-term use of these technologies, and track their accessibility dimensions.

We attempted to include diverse disability perspectives on captioning. However, our sample was limited in size and thus may not capture the multitude of ways captions can support individuals’ access needs, particularly those who are multiply disabled. As stated in Section 6.2.3, being expansive in our conception of caption users is integral. Multiply disabled caption users with unique combinations of access needs, such as those who identify as DeafDisabled or DeafBlind, could further point to ways we rethink captioning practices– for instance, by offering braille access to captions, word bubbling around caption text [98], access to complete transcript, or rapid serial visual presentation [33]. Exploring these could be a valuable space for future work.

Lastly, our interest in multilingual captioning for access meant we focused on spoken and written languages instead of signed languages, as most do not have a standard written form. Exploring individuals’ use of translated captions with sign languages (e.g., English with ASL) could be insightful. Additionally, understanding how individuals navigate interactions with translanguaging between different signed languages, and signed and spoken languages is an interesting avenue of future research.

## 8 Conclusion

We live in a multilingual world. Disabled people speak various languages, and experience varying levels of access needs across different languages. Yet, HCI and accessibility research has implicitly or explicitly focused on English in designing technologies like captioning. Our research contributes a language and disability justice agenda to decenter English, and celebrate all ways of communication. Through stories of 13 multilingual individuals who use captions for accessibility, we show how disabled people navigate multilingual access needs, the roles that multilingual captions play in their lives, and their visions for the future. Overall, our analysis allows us to situate multilingual captioning within broader social, cultural, and linguistic structures. By centering language, decentering fluency, and recognizing fluidity of use, we can orient communication accessibility research toward language and disability justice.

## Figures and Tables

**Table 1:** An excerpt of diary logging instructions sent to participants.

Mode	Activity Type	Example sources	Ways to Log
Online	Media	TV Shows, Movies, YouTube, Instagram reels, TikToks	Share link to media
Online	Video Calls	Video conferencing platforms with monolingual and multilingual automated captioning (ASR)	Name of platform + Screenshot of captions*
Online	Information resources	News, educational lectures	Share link to video or name of particular talk show
In person	Informal conversations	Conversations with friends and family	Short written description of experience or photo/video/Snap/Reel*
In person	Formal conversations	Workplace/professional interactions	Short written description of experience or photo/video/Snap/Reel*
In person	Random	Interactions during errands, day-to-day transactions	Short written description of experience or photo/video/Snap/Reel*

* Participants were told to get consent of conversation partners before taking any photos or recordings.

**Table 2:** Distribution of Languages in our Participant Sample.

Language	Count	Language	Count	Language	Count
Arabic	1	Hokkien	1	Russian	1
ASL	4	Italian	1	Serbian	1
Cantonese	1	Japanese	3	Spanish	4
English	13	Korean	2	Tamil	1
French	5	Malay	1	Ukranian	1
German	2	Mandarin	4	Welsh	1
Hindi	2	Portuguese	2		

The counts represent the number of participants that discussed that language to be a part of their lives.

**Table 3:** Participant Table with multilingual scenarios from diary logs and interviews.

P No.	Languages	Disability	Multilingual experience where they used captions	Multilingual experience where they did not use captions
P1	4+	HoH	Watched Tamil movies with Tamil/English captions; uses transcription app inperson.	Conversation with their nieces where they switch between Hindi and English.
P2	4	HoH	Watched a historical Korean drama with Korean captions; used CART in class.	A call with their sister where they switched between Korean and English.
P3	4	deaf	Watched a Chinese drama with Chinese captions.	A chat with friends where they switched between ASL and spoken Chinese.
P5	4+	ND	Watched news in Arabic from Palestinian journalists with Arabic/English captions.	Played multilingual Scrabble with a friend.
P6	4+	ND	Watching Spanish shows on Netflix with Spanish/English captions.	Taking Spanish classes in college.
P7	4	HoH and ND	Watched YouTube videos in Mandarin with Chinese/English Captions.	Taking Hokkien classes on Zoom.
P8	2	ND	Watching Mandarin media with English captions with their partner.	Conversations in Mandarin with their family.
P9	4	auditory processing, tinnitus	Went to a movie date watching a Japanese movie with English captions.	A conversation with their parents switching between Serbian and English.
P10	3	HoH, chronically ill	Watched TikTok in Hindi with English captions	A dinner with their mother / call with grandmother in Hindi and English.
P11	3	auditory processing	Watched TikToks in French and German with matching captions.	A conversation with their dog in French.
P12	2	disabled	Watches Russian media with Russian/English captions if available.	In person interactions in Russian.
P13	4+	auditory processing	Watched and listened to Japanese karoake videos.	A chat with receptionist / grandma’s nurse in Cantonese and English.

We only list number of languages to protect participant anonymity. Disabilities are characterized as Hard-of-Hearing (HoH), d/Deaf, Neurodivergent (ND), chronic illness, and auditory processing related.

## CCS Concepts

- **Human-centered computing** → **Accessibility**.

## Footnotes

1 P9 highlighted that Serbian/Montenegrin/Bosnian/Croatian are considered mutually intelligible language varieties of Serbo-Croatian and attitudes about language are closely tied to the history of conflict in the region.

2 Chinese macrolanguage: https://www.kevinhsieh.net/2019/02/27/chinese-macrolanguage/

3 A sweet made with carrots

4
https://www.thecanadianencyclopedia.ca/en/article/joual

5 We draw this terminology from Crip Negativity: “What I’m really after is not the availability of access but rather the outcome of access: What are we hoping access will do for us?”[97].

6 Audism is a type of oppression directed at DHH people, privileging hearing senses and ways of communicating [11].
